# Underwater near-infrared spectroscopy can measure training adaptations in adolescent swimmers

**DOI:** 10.7717/peerj.4393

**Published:** 2018-04-20

**Authors:** Ben Jones, Dave Parry, Chris E. Cooper

**Affiliations:** 1 School of Sport, Rehabilitation and Exercise Sciences, University of Essex, Colchester, Essex, UK; 2 Director of Sport, University of Essex, Colchester, Essex, UK

**Keywords:** Adolescents, Near-infrared spectroscopy, Muscle oxygenation, Swimming, Training

## Abstract

The development of an underwater near-infrared spectroscopy (uNIRS) device has enabled previously unattainable measurements of peripheral muscle hemodynamics and oxygenation to be taken within the natural aquatic environment. The purposes of this study were (i) to trial the use of uNIRS, in a real world training study, and (ii) to monitor the effects of a swim training program upon muscle oxygenation status in short distance swimming. A total of 14 junior club level swimmers completed a repeated swim sprint test before and after an eight week endurance training program. A waterproof, portable Near-Infrared Spectroscopy device was attached to the *vastus lateralis*. uNIRS successfully measured changes in muscle oxygenation and blood volume in all individuals; rapid sub-second time resolution of the device was able to demonstrate muscle oxygenation changes during the characteristic swim movements. Post training heart rate recovery and swim performance time were significantly improved. uNIRS data also showed significant changes. A larger rise in deoxyhemoglobin during individual sprints suggested training induced an increase in muscle oxygen extraction; a faster recovery time for muscle oxygenation suggested positive training induced changes and significant changes in muscle blood flow also occur. As a strong correlation was seen between an increased reoxygenation rate and an improved swim performance time, these findings support the use of uNIRS as a new performance analysis tool in swimming.

## Introduction

Near-Infrared Spectroscopy (NIRS) facilitates non-invasive monitoring of changes in the concentration of oxyhemoglobin + oxymyoglobin (O_2_Hb + O_2_Mb) and deoxyhemoglobin + deoxymyoglobin (HHb + HMb) chromophores within the small blood vessels, capillaries and intracellular sites of O_2_ transport and O_2_ uptake (Mancini et al., 1994; Grassi et al., 2003). The growing use of portable NIRS in an applied sport setting has been well documented (Ferrari, Muthalib & Quaresima, 2011; Hamaoka, 2013) and NIRS has provided important insights into the hemodynamic responses that occur during and in response to exercise (Hamaoka, 2013; Perrey & Ferrari, 2017). Beer–Lambert Law derived trend changes in muscle deoxyhemoglobin concentration have been suggested to provide information related to muscle oxygen extraction, whereas spatially resolved spectroscopy (SRS) measures of muscle oxygen saturation (TSI %) have been presumed to inform on the balance between O_2_ delivery and O_2_ consumption within the muscle (Buchheit & Ufland, 2011; Boushel et al., 2001). Measures of the change in total muscle hemoglobin concentration (tHb) have the potential to inform on changes in blood flow (Hesford et al., 2012).

As a completely non-invasive optical method with strong signal to noise ratio and millisecond time resolution NIRS is readily translatable to extreme and challenging environments. For example, NIRS has previously been used in both indoor speed skating (Hesford et al., 2013) and outdoor biathlon (Hesford, Laing & Cooper, 2013). However, to date studies in extreme exercise environments have been limited. One area of obvious interest to exercise scientists where real time physiological measures in the field are challenging to undertake is water-based activities. Yet in principle there is no reason why muscle NIRS measurements should not be transferable to all aquatic environments.

The main challenge of underwater NIRS is preventing light piping from source to detector via the waterproof material employed. However, we recently developed, tested and validated a waterproof adaptation of a commercial portable NIRS spectrometer (PortaMon; Artinis Medical Systems, BV, The Netherlands) (Jones, Dat & Cooper, 2014). We chose to examine swimming as this is a popular exercise mode (Koenig et al., 2014) with currently limited physiological measurement tools.

Adolescent (12–18 years) swimmers present an interesting research population as little “in water” research has been conducted within this group. Longitudinal adult studies (Costa et al., 2012a; Perini et al., 2006) have demonstrated that during a swimming competition season, physiological measures such as heart rate and swim performance will vary among athletes and that the greatest degree of change is usually seen within the early part of the training season (Costa et al., 2012b). During this period, high volume, aerobic endurance training is usually chosen by swim coaches (Aspenes & Karlsen, 2012) with the two major goals focusing upon improving stroke efficiency and establishing a strong aerobic base (Maglischo, 2003). Contention surrounds this type of training (Costa et al., 2012a), as most individual competitive swimming events last less than 3 min (Libicz, Roels & Millet, 2005). Therefore, this type of training seemingly does not match the energetic demands of competitive races. Current methods utilized to monitor training response during and following swim programs commonly involve the use of heart rate (HR) data, blood lactate profiling and session rating of perceived exertion (RPE) (Keskinen, Keskinen & Mero, 2007; Pyne, Lee & Swanwick, 2001; Rodríguez-Zamora et al., 2012). These indices are used in part as they are simple to implement and user friendly, but also because there is a lack of developed, swimming-specific, technology available for physiological assessment. This highlights the need for a non-invasive, easy to use technology such as underwater near-infrared spectroscopy (uNIRS) that may help to evaluate physiological monitoring within the swim environment.

The purposes of this study are (i) to trial uNIRS, in a repeated measures training study and (ii) to monitor the effects of a swim training program upon muscle oxygenation status within the swim athlete’s primary training environment. It was hypothesized that the uNIRS device would obtain hemodynamic data that would inform upon swimmers’ responses to an aerobic endurance training program, specifically; following training, significant changes in both heart rate and uNIRS monitored variables indicative of an improved oxidative metabolism would be evident with a concomitant improvement in swim performance.

## Methods

### Ethics statement

All participants and their parents/guardians gave their written informed consent before taking part in the study which was approved by the ethics committee of the University of Essex.

### Experimental overview

Fourteen junior club-level swimmers were assessed from three different swimming programs within the Essex (UK) area (nine male and five female): age 15.3 ± 1.7 years, height 171 ± 9.2 cm, weight 64.2 ± 11.0 kg, quadriceps skin fold 15.6 ± 5.2 mm. Females reported a non-significant (*p* = 0.08) higher skinfold (18.8 ± 5.1 mm) than males (13.6 ± 4.4 mm). Two participants were subsequently excluded from data analysis. Participants completed 5 × 100 m maximal freestyle exercise interspersed with 3 min recovery periods before and after an eight-week high volume endurance training period. All participants were sprint distance swimmers (<200 m). A waterproof, portable NIRS device was attached to the dominant leg, *vastus lateralis* to provide a non-invasive measure of muscle oxygenation throughout the swim test (Jones, Dat & Cooper, 2014). Time taken to complete each 100 m sprint (s), HR, and subjective RPE 6–20 Borg scale (Borg, 1982) were also recorded. Each subject’s height and body mass was collected using a wall mounted stadiometer (220; Seca, Birmingham, UK) and electronic scales (770 digital; Seca, Birmingham, UK). Thickness of the adipose layer of tissue overlying the quadriceps muscle was measured at the midpoint between NIRS optodes, using a skinfold caliper (British Indicators, Harpenden, UK) and no significant differences between baseline and post-training descriptive measures were found (*p* = 0.42). Sexual maturity was assessed by self-report using the indices of pubic hair described by Tanner (Tanner, 1962). All children reported a maturity level of three to four indicating the study population was pubertal. Participants completed the physical activity readiness questionnaire for everyone (PAR-Q+) and were informed that they could remove themselves from the study at any time point.

### Experimental procedures

All swim training and testing took place in the short course training pool (25 m) of the participants club, with a pool temperature between 28 and 30 °C. All trials took place at the same time of day before practice. All swim tests started in the water, pushing off from the wall. Participants were allowed to tumble turn and were timed manually using a handheld stop watch (Fastime DB1; AST LTD, Leicester, UK) for 25 m split times and final 100 m time (s). All participants were given strong verbal encouragement to give maximum effort during each 100 m repetition. Participant’s completed a standardized warm up prior to testing which consisted of 800 m of mixed strokes and variable pace swimming, administered by the swim coach. Following the warm up, participants were required to remain upright with their body weight evenly distributed for 3 min in order to establish a baseline measurement. The repeat swim test consisted of 5 × 100 m maximal freestyle swim exercise. Each repetition was interspersed with a 3 min recovery period, during which participants were required to stand upright, with their weight evenly distributed over each leg. During testing, HR was monitored using a Polar (Polar FT2™; Polar Electro Oy, Kempele, Finland) wireless chest strap. HR was recorded immediately post-sprint and also one and 2 min after each 100 m effort. Heart rate recovery (HRR) was considered to be the difference between End Exercise HR (HR_EE_) and HR at the end of the 2 min. Participant RPE was taken immediately post-sprint with participants indicating by pointing to a level on the 6–20 Borg scale (Borg, 1982). All testing measures were completed both pre and post-training intervention.

### Swim repeated exercise test

The rationale of choosing a repeat swim test was based upon; (i) all the junior swimmers being sprint specialists; (ii) participants having no prior experience of standardized aerobic endurance tests and; (iii) a large aerobic contribution has been shown during swimming exercise between 30 s and 4 min (Costa et al., 2012b). Additionally land based maximal intensity exercise tests (4 × 30 s with 4 min rest) have demonstrated contributions from both anaerobic and aerobic metabolism (Wittekind & Beneke, 2011). In further support of the testing sequence similar repeat sprint distance tests (6 × 50 m) have been employed in young swimmers (Kabasakalis et al., 2014) and repeat sprint tests have been suggested as an appropriate testing procedure for sprint swimmers (Maglischo, 2003).

### Training intervention

Swim training mostly consisted of five sessions in the pool each week, involving a mixture of low, moderate, and high intensity aerobic training and short interval sprint work. Technical training was additionally performed during low intensity aerobic tasks. Weekly training volume averaged 35 ± 8 km week^−1^ and training attendance was monitored by swim coaches. One participant was unable to complete the post-training measurement due to injury and one participant completed less than half the required training intervention. All participants included within data analysis completed more than 95% of the training sessions.

### Near infrared spectroscopy measurements

During all swim testing, a specially waterproofed portable NIRS apparatus (Jones, Dat & Cooper, 2014) (PortaMon; Artinis Medical Systems, BV, The Netherlands), was positioned on the (dominant limb) belly of the *vastus lateralis* muscle, midway between the greater trochanter of the femur and the lateral femoral epicondyle. The chosen site for NIRS placement was based on a previous application (Jones & Cooper, 2016) in swim athletes. To ensure the optodes and detector did not move relative to the subject’s skin, the device was fixed into position using sports waterproof adhesive tape and secured using the participant’s own specialist swim apparel. All participants wore swim apparel that consisted of knee length compressive fabric swimsuits that aided in device fixation. A surgical marker pen was used to mark probe placement in order to identify any device movement during testing. No sliding was observed at the end of any measurement in any subject. To ensure accuracy of repeat device placement, an outline of the device was drawn onto each subject’s leg. Subjects were asked to maintain the outline during the testing period. Additionally, any bodily hair within the probe placement area was removed from each subject’s skin and subjects were asked not to use any moisturizing products on the testing day. The same researcher attached each device and applied the sports strapping. In order to account for the external pressure applied to the muscle belly a standardized approach (i.e., single fold strapping) was utilized. Subject’s reported that the device did not interfere with their normal swim action.

Using the PortaMon device (750 and 850 nm two wavelength continuous wave system) it is not possible to separate the changes in light absorbed by hemoglobin or myoglobin. Furthermore the relative contribution of hemoglobin and myoglobin to changes in the NIRS signal is controversial (Davis & Barstow, 2013; Seiyama, Hazeki & Tamura, 1988). As the total muscle myoglobin content does not change in the course of an acute study, we therefore uses the modified Beer–Lambert law to report the NIRS data as changes in tissue oxyhaemoglobin + oxymyoglobin (O_2_Hb+O_2_Mb), tissue deoxyhaemoglobin + deoxymyoglobin (HHb + HMb), total tissue haemoglobin (tHb) and SRS method (Patterson, Chance & Wilson, 1989; Suzuki et al., 1999) measured tissue oxygenation (TSI %) (Jones, Hamilton & Cooper, 2015). In the present study, chromophore concentrations were detected using the furthest light-emitting diode (40 mm). Values for O_2_Hb+O_2_Mb, HHb + HMb, and tHb are reported as a change (Δ) from baseline (30-s averaging before each test) in micromolar units (μM.cm). The Δ calculated during swim exercise is the difference between the 30 s baseline average and the average of the 3 s surrounding the minimum desaturation value (excluding tumble turn data). Δ values obtained during recovery periods were taken as the difference between the baseline value and the 3 s average of the maximum value achieved during the recovery period. During testing, the PortaMon stored the data within the device’s internal memory capacity. This data was subsequently downloaded onto a personal computer for data analysis through an online software program. Data acquisition was sampled at a rate of 10 Hz for analogue-to-digital conversion and subsequent analysis.

### NIRS data analysis

The recovery kinetics of TSI (%) could not be modelled utilizing an exponential function with a time delay as described in previous studies (Buchheit et al., 2012) as the TSI (%) did not approximate a clear exponential response in this subject group. Similarly, the HHb + HMb signal following dynamic exercise has previously been shown (Buchheit et al., 2011) to be difficult to model and a minimum fit criterion of data acceptance was set (*r*^2^ = >0.90); this led to the subsequent exclusion of most subject’s TSI (%) data and the majority of their HHb + HMb signal. As a result, HHb + HMb recovery data was not included in our subsequent analysis. We therefore chose to utilize a simpler phenomenological measure of reoxygenation rate (reoxy rate) (% s^−1^) calculated as the change in TSI (%) from the end of the sprint by fitting a linear model to the initial part of the TSI (%) recovery (first 40 s) vs. time. The slope of the relationship was retained as an index of reoxygenation rate (reoxy rate % s^−1^).

## Statistical Analyses

Descriptive statistics are presented as a mean ± SD unless otherwise stated. Each variable was examined with the Kolmogorov–Smirnov normality test. For pre- vs. post-training comparisons, all NIRS variables, HR, and swim times were analyzed using either paired (2-tailed) *t*-tests or Wilcoxon signed rank test when the sample normality test failed as per Bravo et al. (2012). The level of significance for analyses was set at *p* < 0.05. The standardized difference or effects size (ES) in each performance measure was calculated using the pooled standard deviation. Data was also assessed for clinical significance using an approach based on the magnitudes of change with 95% confidence limits (95% CL) (Hopkins et al., 2009). Pearson’s product–moment correlation analysis was used to compare association between optical measures and performance. All analyses were performed using Graphpad Prism 6 (Graphpad Software, San Diego, CA, USA).

## Results

### Underwater near-infrared spectroscopy

**Figure 1:**
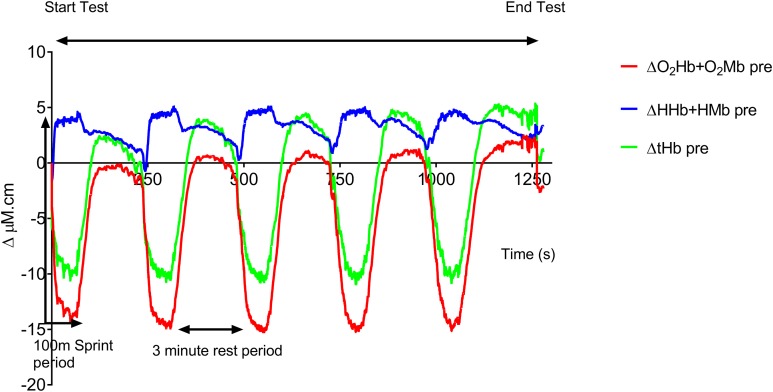
Group mean pre-training hemodynamic responses during the repeat swim test.

Figure 1 shows the averaged group data before training. The following trends in this group data were also seen in all the individual traces. At the onset of each 100 m sprint there is a rapid drop in both O_2_Hb + O_2_Mb and tHb and a concomitant rise in HHb + HMb amplitude. O_2_Hb + O_2_Mb and tHb reach a plateau after ∼20 s during each swim sprint, whereas HHb + HMb amplitude continues to rise throughout the swim period, with a peak occurring at the end of the 100 m. During the recovery period there is a steady rise in both O_2_Hb + O_2_Mb and tHb with a reciprocal drop in HHb + HMb. O_2_Hb + O_2_Mb recovers to baseline values after each sprint, whereas tHb displays an incremental hyperemic response after each sprint effort.

**Figure 2:**
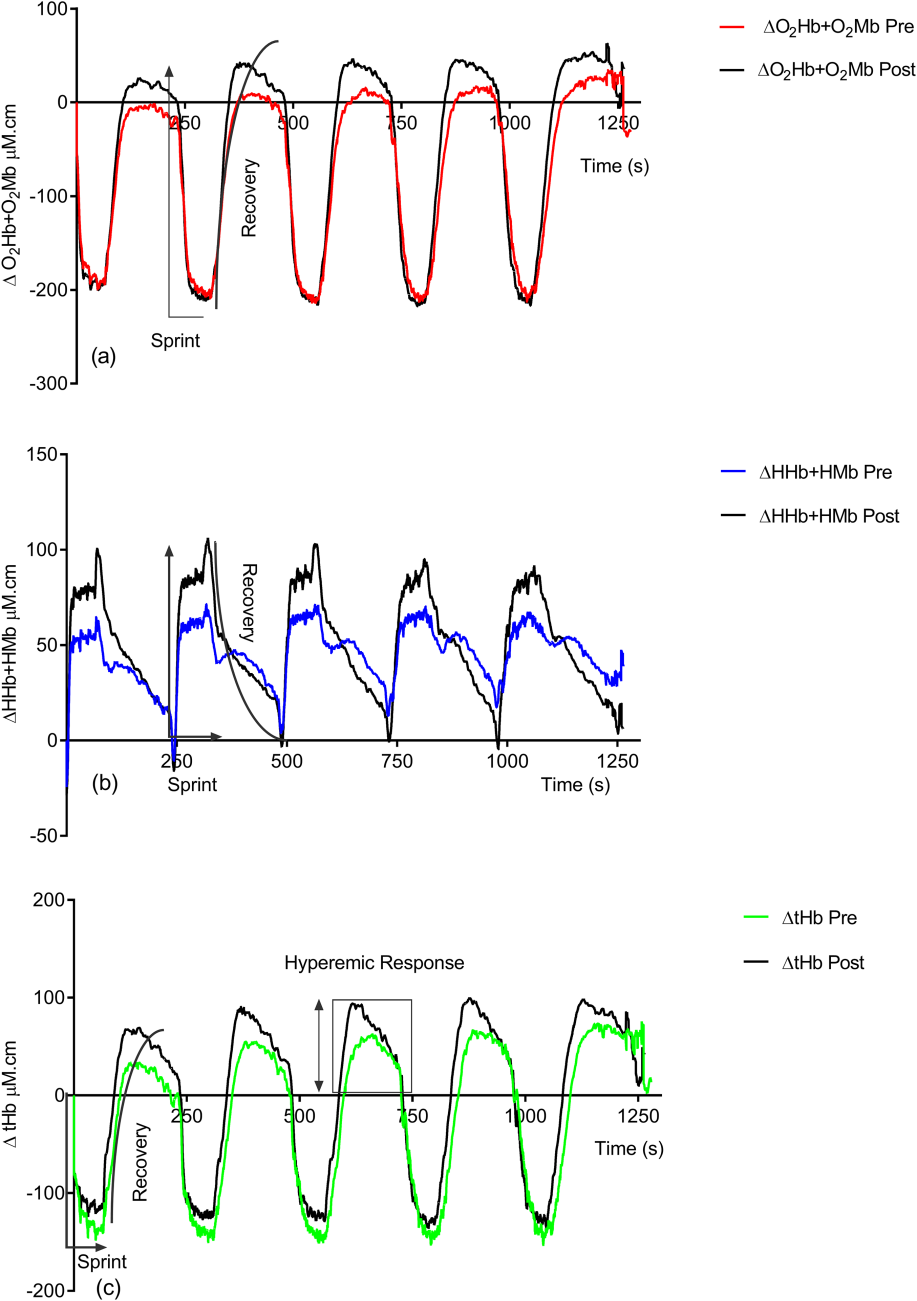
Group pre vs. post-training hemoglobin overlay traces during the entire repeat swim sprint tests; (A) O2Hb + O2Mb, (B) HHb + HMb and (C) tHb.

Figure 2 shows the effect of training on the observed NIRS changes throughout the study. Significant changes are seen in both the sprint and recovery phases. Table 1 quantifies these for all individuals during the sprint efforts and during the recovery phase. There is a significant group increase in the deoxyhemoglobin + deoxymyoglobin (ΔHHb + HMb) concentration (ES = 0.4, 95% CL = −0.4–1.2; *p* = 0.04) post-training; this is also reflected in the individual data, with only two subjects showing a decrease. There is no significant change in oxyhemoglobin + oxymyoglobin (ΔO_2_Hb + O_2_Mb) (ES = 0.3, 95% CL = 0.4–1.1; *p* = 0.12) or total hemoglobin (ΔtHb) (ES = 0.08, 95% CL = −0.7–0.8; *p* = 0.78).

**Table 1:** Individual (pre vs. post-training) changes in oxyhemoglobin + oxymyoglobin (ΔO_2_Hb + O_2_Mb), deoxyhemoglobin + deoxymyoglobin (ΔHHb + HMb) and total hemoglobin (ΔtHb) during swim and recovery efforts (*p* < 0.05).

Subject	Pre ΔO_2_Hb + O_2_Mb	Post ΔO_2_Hb + O_2_Mb	Pre ΔHHb + HMb	Post ΔHHb + HMb	Pre ΔtHb	Post ΔtHb	Pre Recovery ΔO_2_Hb + O_2_Mb	Post Recovery ΔO_2_Hb + O_2_Mb	Pre Recovery ΔtHb	Post Recovery ΔtHb
**S1**	−154.42	−154.84	68.04	73.92	−105.56	−92.12	64.96	103.40	104.08	163.10
**S4**	−179.06	−183.26	44.24	88.62	−136.36	−104.44	76.24	76.36	136.89	160.47
**S5**	−170.66	−174.58	95.90	95.20	−112.28	−108.36	−9.60	54.07	57.60	132.24
**S6**	−133.56	−141.96	0.98	16.66	−149.38	−151.2	76.41	87.28	138.32	118.92
**S7**	−142.94	−129.78	−7.98	−1.12	−168.70	−142.24	64.34	26.29	114.49	61.21
**S8**	−179.90	−184.80	−7.98	44.38	−184.94	−145.6	−4.68	40.04	5.88	82.66
**S9**	−171.08	−170.66	106.68	90.30	−78.12	−88.06	118.47	145.26	165.42	164.86
**S10**	−162.54	−180.18	4.34	31.36	−174.72	−158.34	−136.14	−106.18	−127.06	−92.04
**S11**	−111.16	−242.62	61.60	116.20	−52.92	−140.00	−12.26	−20.05	52.72	30.63
**S12**	−277.06	−447.72	210.42	422.80	−83.86	−82.74	47.68	92.65	123.98	211.12
**S13**	−105.00	−140.14	11.90	70.00	−99.68	−143.92	65.04	95.98	104.61	135.16
**S14**	−285.32	−266.98	202.86	204.82	−171.78	−125.02	82.18	110.66	141.96	190.32
**Mean ± SD**	−172.73 ± 56.28	−201.46 ± 87.36	65.92 ± 73.33	104.43 ± 108.79*	−126.56 ± 43.82	−123.48 ± 27.02	36.05 ± 67.76	58.81 ± 67.87*	84.91 ± 80.57	113.22 ± 83.33*

**Note:**

* Denotes significance when compared to pre-training value (*p* < 0.05).

Following training there is significant increases in ΔO_2_Hb + O_2_Mb (ES = 0.3, 95% CL = −0.4–1.1; *p* = 0.02) (pre = 36.05 ± 67.76 μM.cm vs. post = 58.81 ± 67.87 μM.cm) and ΔtHb (ES = 0.3, 95% CL = −0.4–1.1; *p* = 0.04) (pre = 84.91 ± 80.57 μM.cm vs. post = 113.22 ± 83.33 μM.cm) during the recovery phase.

**Figure 3:**
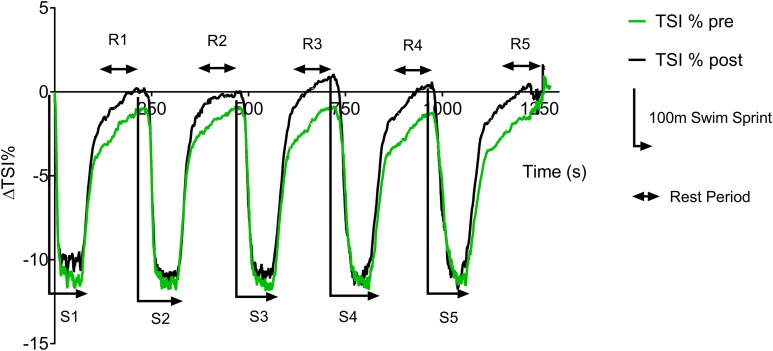
Pre vs. post-training ΔTSI (%) during repeat swim test.

Figure 3 displays the group average pre vs. post-training ΔTSI (%) data trace during swim testing. During the swim test, there is a rapid drop in TSI at the start of each sprint, with a plateau occurring ∼20 s into each 100 m sprint. During the 3 min recovery period there is a rapid recovery of TSI during the initial part of the recovery, followed by a slower recovery during the second and third minute. However, group TSI (%) fails to return to baseline values during the pre-training test, whereas a complete recovery is found post training.

Table 2 displays the individual pre vs. post-training ΔTSI (%) and calculated reoxygenation rates (%.s^−1^). A non-significant post-training increase in the extent of desaturation (ΔTSI %) in the group mean (ES = 0.17, 95% CL = −0.6–0.9; *p* = 0.54) can be seen; this was largely reflected in the individual data. A non-significant increase was seen in the group mean post-training reoxygenation rate (ES = 0.7, 95% CL = −0.07–1.5; *p* = 0.27).

**Table 2:** Pre vs. post-training ΔTSI (%) and TSI recovery kinetics.

Subject	Pre ΔTSI (%)	Post ΔTSI (%)	Pre reoxy rate (%.s^−1^)	Post reoxy rate (%.s^−1^)
**S1**	−9.08	−11.07	0.18	0.21
**S4**	−12.85	−12.9	0.19	0.20
**S5**	−6.22	−6.44	0.31	0.25
**S6**	−15.99	−12.64	0.21	0.19
**S7**	−7.97	−8.1	0.11	0.09
**S8**	−8.99	−10.83	0.15	0.21
**S9**	−14.69	−11.92	0.29	0.26
**S10**	−7.58	−10.15	0.10	0.21
**S11**	−10.63	−16.52	0.17	0.32
**S12**	−17	−19.41	0.34	0.42
**S13**	−6.74	−7.12	0.16	0.16
**S14**	−16.63	−13.17	0.31	0.26
**Mean ± SD**	**−11.45 ± 4.24**	**−12.20 ± 4.14**	**0.21 ± 0.08**	**0.23 ± 0.08**

**Figure 4:**
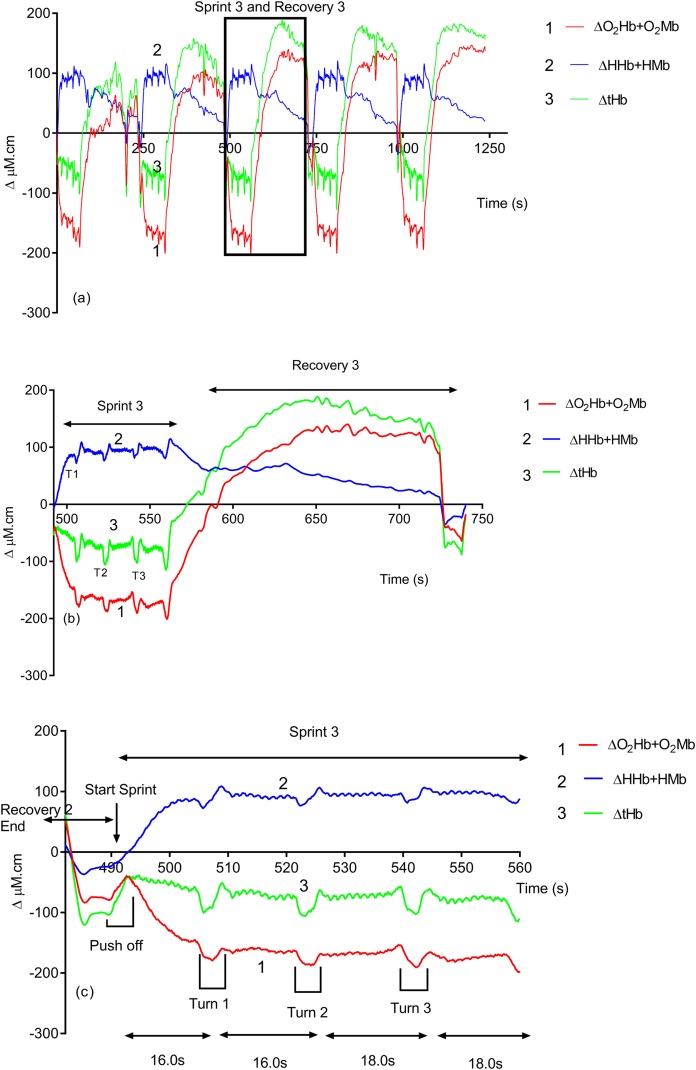
An individual subject’s hemoglobin concentration changes displaying the entire sprint (A) and a single swim sprint on enhanced scales (B and C).

**Figure 5:**
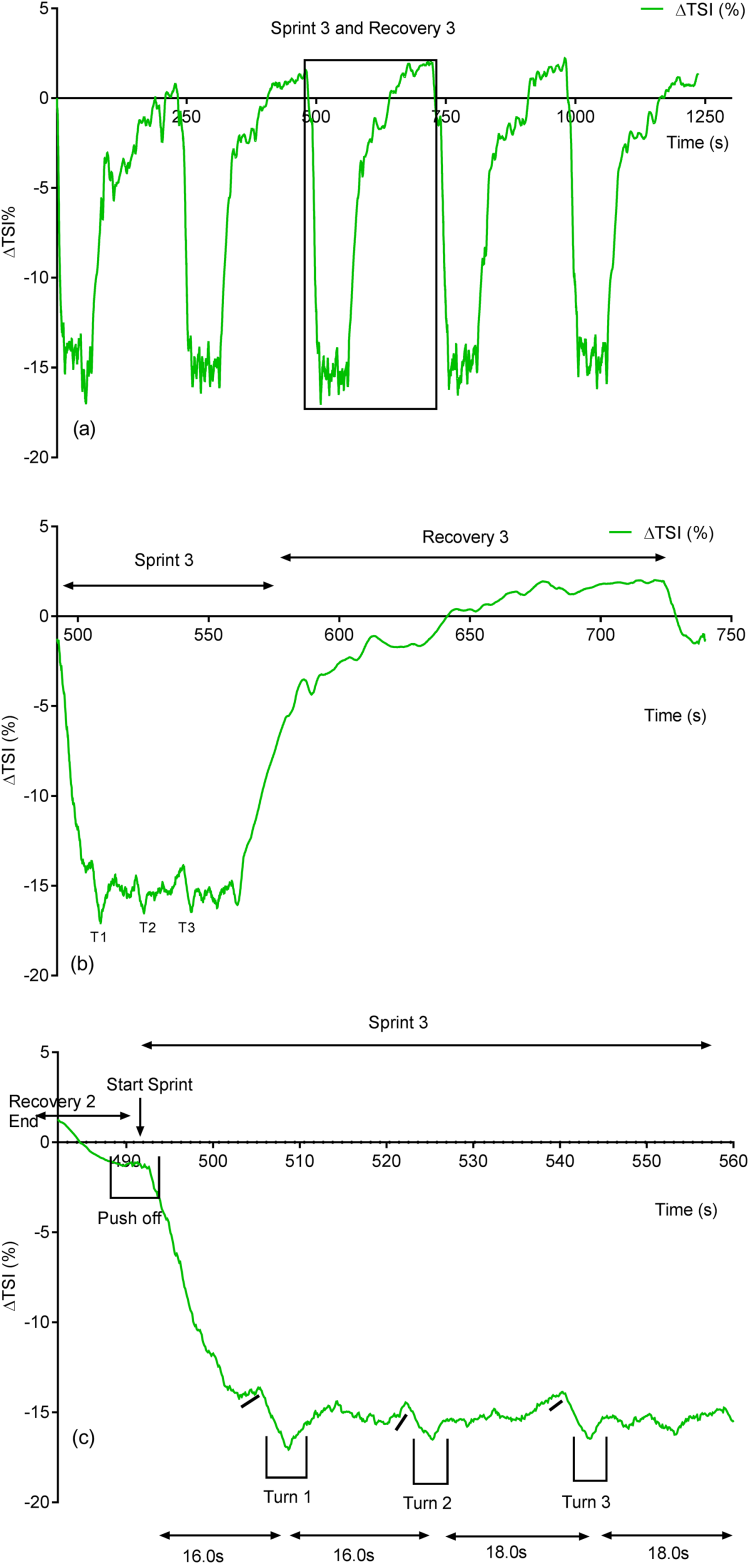
Individual subject’s tissue saturation index (TSI %) trace displaying the entire sprint (A) and a single swim sprint plotted on enhanced scales (B and C).

Due to its rapid time resolution uNIRS is able to report on changes in oxygenation and blood volume during changes in swim biomechanics. This is illustrated in Figs. 4 and 5. Figure 4 displays the hemoglobin concentration changes for a representative subject (the sprint three and recovery three traces are plotted on an enhanced scale). Figure 4 is representative of the group data and exhibits a drop in O_2_Hb + O_2_Mb and tHb during the sprint period, with a concomitant rise in HHb + HMb. The recovery period mirrors these changes with an increase in O_2_Hb + O_2_Mb/tHb and a drop in HHb + HMb. Figure 5 displays the same data for the subject’s tissue saturation index.

Clear uNIRS changes are seen when the subject pushes off the wall at the start of the sprint and when the subject turns at the end of each 25 m length. Broadly speaking where a biomechanical event occurs that is expected to constrict the muscle a drop in tissue oxygenation and blood volume can be seen. This is consistent with a drop in muscle blood flow, the drop in volume may also be due to a shortening in muscle length decreasing the volume of tissue interrogated by the light; however, this change would be unlikely to significantly decrease tissue oxygenation as well, hence our preference for an explanation based on blood flow changes. The grey dash line just below the TSI (%) trace indicates where the subject ceases their leg kicking (slight resaturation and tHb increase can be seen) action and glides into the wall. As the subject completes the tumble turn action (somersault), the legs are tucked into the body (hips flexed ∼90°), which results in a short occlusion, which is evidenced by the further drop in TSI (%) and tHb during each turning action. Following each turn the subject pushes off from the wall and there is a resaturation and tHb increase as the subject’s hip and knees are extended, the subject then resumes the kicking action and the TSI (%) plateaus.

### ΔHeart Rate Recovery and ΔSwim Performance

Table 3 reports on the global physiological, psychological and performance characteristics of the athletes. Significant post training group changes were seen in %ΔHHR (ES = 0.9, 95% CL = 0.02–1.7 *p* = 0.003) and Swim Performance (SP) time (ES = 0.4, 95% CL = −0.3–1.2; *p* = 0.01). Individual data showed that nine subjects showed increased HHR responses post-training, with two subjects displaying decreased HRR. Nine subjects displayed improved swim performances, with three subjects producing slower repeat sprint times post-training. Non-significant post-training changes were seen in subject RPE (ES = 1.4, 95% CL = 0.5–2.2; *p* = 0.31).

**Table 3:** Pre vs. post-training heart rate recovery percentage change (%ΔHR), pre vs. post-training individual swim performance time (s) and pre vs. post-training individual rating of perceived exertion (RPE) 6–20 Borg scale.

Subject	Heart rate recovery (HRR %Δ)		Swim performance (s)		RPE (6–20)	
	PRE	POST	PRE	POST	PRE	POST
**S1**	−38.58	−37.63	67.4	67.2	18	16
**S4**	−33.33	−33.90	68.6	67.0	17	18
**S5**	−21.71	−25.96	71.4	69.0	16	17
**S6**	−26.01	−38.37	72.4	72.8	17	15
**S7**	−20.52	−29.47	71.4	70.8	17	15
**S8**	−27.11	−30.85	71.6	69.4	19.	19
**S9**	−26.21	−31.66	67.4	65.8	17	18
**S10**	NA	NA	68.4	64.6	18	19
**S11**	−21.91	−26.73	75.8	71.8	19	18
**S12**	−31.03	−38.15	64.8	65.2	19	20
**S13**	−28.88	−29.50	68.8	68.4	18	17
**S14**	−29.69	−31.70	67.6	67.8	19	17
**Mean ± SD**	**−27.73 ± 5.41**	−32.18 ± 4.37*	**69.63 ± 2.96**	68.32 ± 2.58*	**18 ± 1**	**17 ± 2**

**Notes:**

Heart rate data was unable to be collected for subject S10.

* Denotes significance when compared to pre-training value (*p* < 0.05).

**Figure 6:**
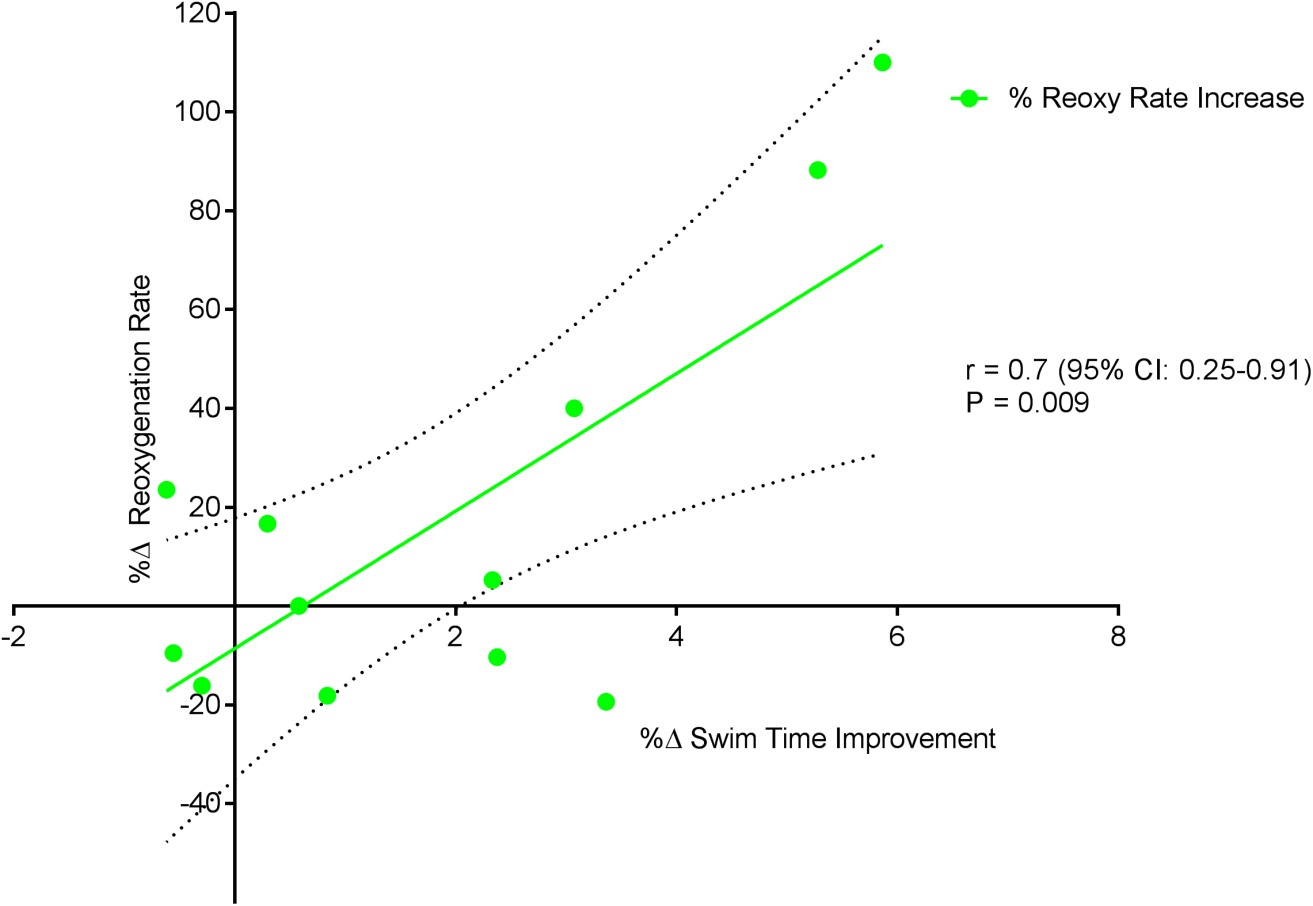
Significant positive relationship between %Δ reoxygenation rate increase and %Δ swim time improvement. Dotted lines are 95% confidence intervals.

**Figure 7:**
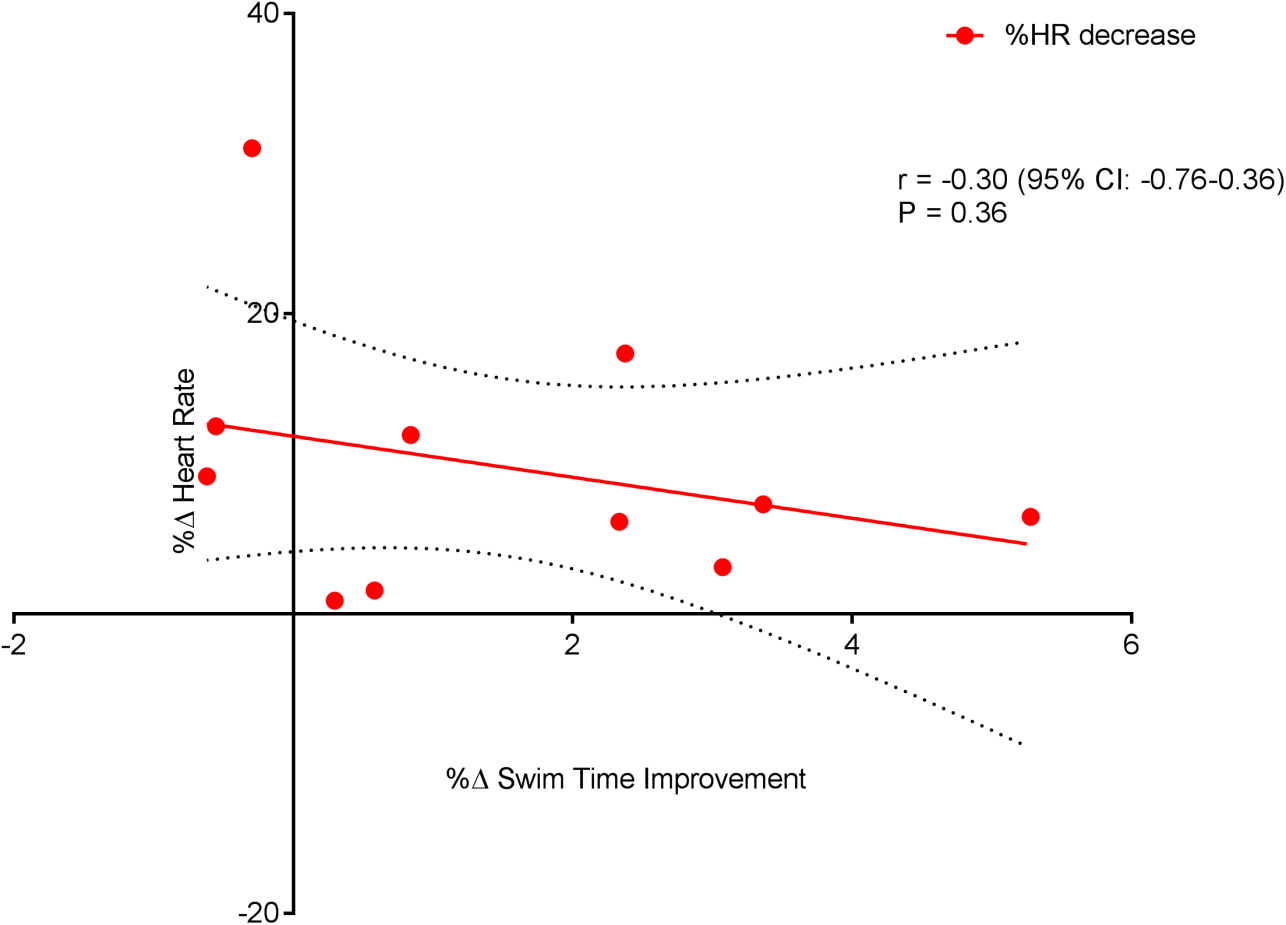
Non-significant negative correlation between %Δ heart rate decrease and %Δ swim time improvement. Dotted lines are 95% confidence intervals.

**Figure 8:**
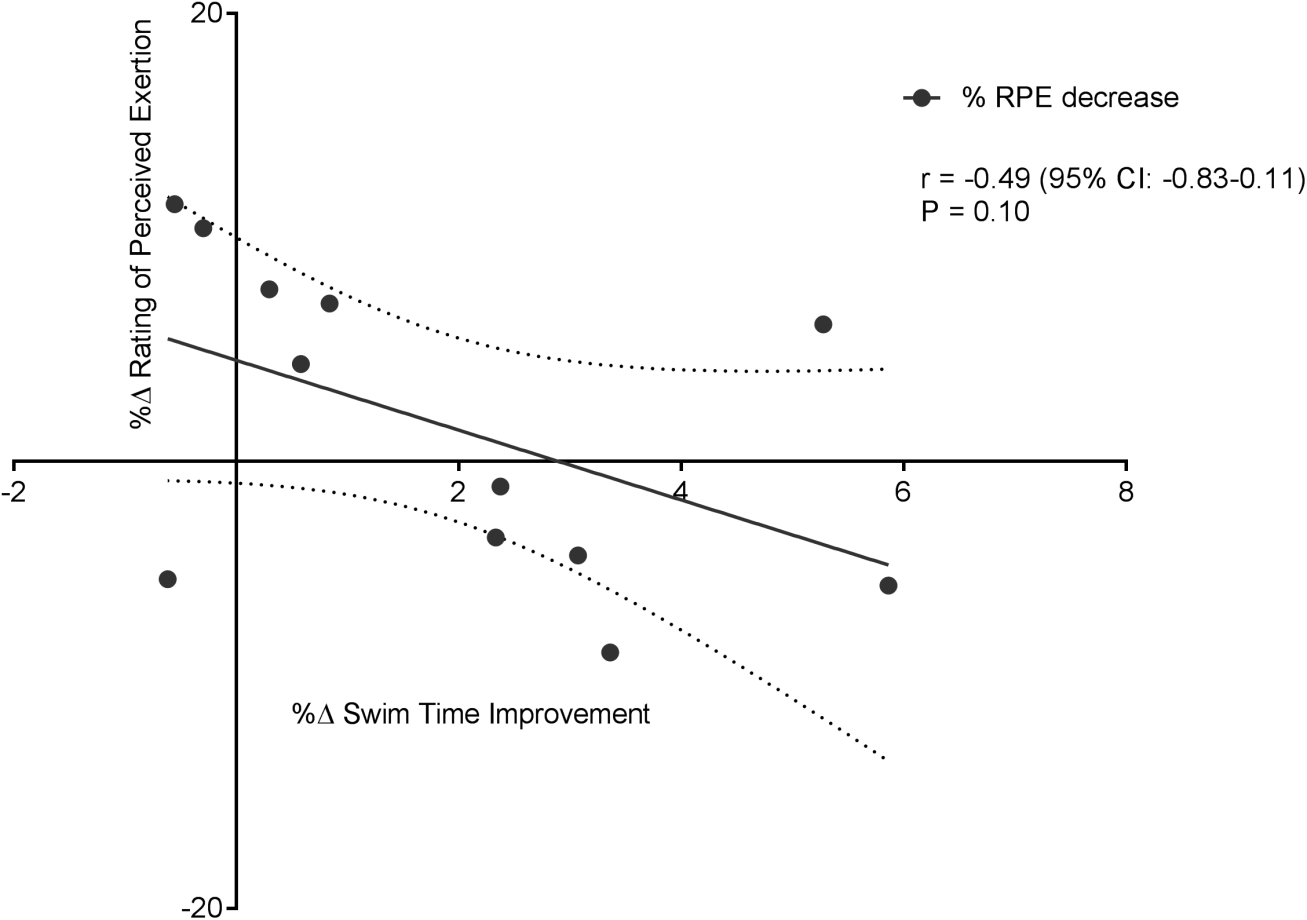
Non-significant negative correlation between %Δ rating of perceived exertion decrease and %Δ swim time improvement. Dotted lines are 95% confidence intervals.

Figure 6 displays a significant positive correlation between Δ% swim time improvement and Δ% reoxy rate increase (*r* = 0.72; 95% CL = 0.25–0.92; *p* = 0.007). Figures 7 and 8 display non-significant negative correlations between Δ% swim time improvement and Δ% HR decrease (*r* = −0.30, 95% CL = −0.76–0.37; *p* = 0.36) and between Δ% swim time improvement and %Δ RPE decrease (*r* = −0.49, 95% CL = −0.83–0.11; *p* = 0.10).

No significant correlations were evident between swim time improvement (SP) and other NIRS measures, nor between HR and NIRS measures; %ΔTSI vs. %ΔSP; *r* = 0.54, 95% CL = −0.05–0.85; *p* = 0.06; %ΔHHb vs. %ΔSP; *r* = 0.06, 95% CL = −0.53–0.61; *p* = 0.85; and %ΔHR vs. %ΔTSI; *r* = 0.04, 95% CL = −0.57–0.62; *p* = 0.89; %ΔHR vs. %ΔHHb; *r* = 0.43, 95% CL = −0.23–0.82; *p* = 0.18.

## Discussion

The purpose (s) of this study were: to trial the ability of uNIRS to monitor the effects of an eight week aerobic endurance (END) training program upon muscle oxygenation status, HR and SP. The portable uNIRS device successfully monitored peripheral muscle hemodynamic responses during both pre and post-training exercise tests, with no loss of data. Furthermore, novel real time physiological data was acquired on a sub second time scale enabling real time visual comparisons between biomechanical movements and muscle oxygen physiology. Such comparisons have proved beneficial in understanding the link between biomechanics and physiology in short track speed skating (Hesford et al., 2012); this study extends this ability to underwater exercise modalities.

uNIRS data revealed a significant post-training, group increase in HHb + HMb amplitude and non-significant increase (desaturation) in ΔTSI (%). HR data which is representative of a central index of the cardiovascular system response to END training, displayed a significantly improved mean recovery rate; group SP was also significantly improved (reduced) post-training. Therefore, in line with our hypothesis, significant changes in both systemic and peripheral physiological indices occurred in response to an END training program with a significant concurrent improvement in swim performance. Interestingly there was a strong correlation between the increase in recovery of muscle oxygenation and improved swim performance time.

### uNIRS assessment

Underwater near-infrared spectroscopy data showed a post-training significantly increased HHb + HMb amplitude and non-significant increase in TSI % desaturation. TSI (%) and HHb + HMb Near Infrared Red (NIR) signals are suggested to provide an estimation of arteriovenous oxygen difference (a-vo_2diff_) i.e., oxygen extraction (Boushel et al., 2001; Subudhi, Dimmen & Roach, 2007; Boone et al., 2012). The significant increase in HHb + HMb amplitude and increase in TSI (%) desaturation seen in this study most likely reflects a training induced enhancement in oxygen extraction capability (Bailey et al., 2009; Prieur & Mucci, 2013). NIRS use within adolescent swim populations has previously solely focused upon the identification of physiological differences related to training and maturity status (McNarry & Jones, 2014) and not the differences seen following a specific training intervention. The current study indicates that uNIRS has the ability to identify peripheral muscle oxygenation adaptations to exercise within adolescent populations.

A possible explanation for the strong association between Δ% reoxy rate and Δ% swim time improvement may relate to the relative importance of muscle oxygen availability and its replenishment during repeat swim sprint performance. Given the lack of association between swim time improvement, systemic (HR) and perceptual (RPE) markers of improved performance, it appears that reoxy rate and uNIRS can aid physiological assessment in swim athletes. An increase in reoxy rate (%.s^−1^) points toward a more rapid increase in oxygen recovery following exercise cessation (Buchheit et al., 2012; Buchheit, Laursen & Ahmaidi, 2009) potentially as a result of an increased rate of muscle blood volume/flow, increasing the rate of oxygen delivery, replenishing energy substrates and removing inhibitory metabolites (Glaister, 2005). The increased hyperemic response (tHb, Fig. 2C) post training supports this increased blood volume/flow hypothesis.

Whilst the availability of, and ability to recover, muscle oxygen is a key factor in recovery between sprints, it is neuromuscular efficiency (i.e., motor unit recruitment, conduction velocity) which is considered to be the primary determinant of the individual and repeat sprint performance (Buchheit & Ufland, 2011; Bishop, Girard & Mendez-Villanueva, 2011). The presence of a plateau in the TSI signal that often occurs during maximal intensity exercise (present in the current study) is interpreted as evidence of maximal extraction capacity (Billaut & Buchheit, 2013). As subjects were able to continue maximal intensity exercise after the appearance of this plateau, it is suggested that perhaps the level of muscle deoxygenation that occurred during testing does not limit continued exercise performance. This assessment is supported by Subudhi, Dimmen & Roach (2007) who showed continued exercise capacity after maximal *vastus lateralis* desaturation during incremental cycle exercise. Evidence from the current study suggests that END training has a significant effect upon the oxidative metabolism of the muscle; and this improved ability is strongly associated with the improved exercise performance. This has implications for the use of uNIRS as a performance analysis tool when studying the effects of high volume distance training in sprint distance adolescent swimmers.

### uNIRS turn data

Novel and interesting findings were seen in both hemoglobin and muscle oxygen traces (Figs. 4 and 5) during swim turns. Visual interpretation of this indicates that a clear constriction occurred during the swim turning action, resulting in a drop in blood volume. A wealth of previous NIRS data report that the physical occlusion of muscle can cause the sort of changes in the total hemoglobin concentration that we see in this study. The importance of the turning phase in total race performance has been well documented (Pereira et al., 2015) and studies have shown that subtle changes in turning action can improve swim performance (Araujo et al., 2010; Veiga et al., 2014; Pereira et al., 2015). Current optimization of the swim turn action has focused on video, surface electromyography (sEMG) and underwater force platform analysis. Swim velocity into turn, peak force and knee angle have been identified as playing a significant role in relation to turn performance (Araujo et al., 2010) uNIRS could provide information in support or contradiction of kinematic analysis with regard to turn maximization and the subsequent underwater swim phase. The use of NIRS in combination with sEMG has been suggested to provide complementary information for muscle activity monitoring (Guo et al., 2014). The recent development of a wireless, waterproof sEMG device (Martens et al., 2015) will enable this line of analysis.

### SP time and HRR assessment

Swim Performance, assessed as the average 100 m (5 × 100 m) swim time(s), significantly improved (*p* = 0.01). The improvement in repeat sprint SP is indicative of an improved aerobic metabolism, given that the contribution of aerobic metabolism to repeat sprint exercise is considerably increased during subsequent sprint efforts (Spencer et al., 2005). However, single best sprint performance was unchanged (pre = 67.08 ± 2.08 vs. post = 67.08 ± 2.75) post-training. Previously, high volume swim training (∼10 km per day) has been shown to reduce single sprint time in collegiate male swimmers (Costill et al., 1991). However, the daily training distances within the current study (∼7 km) were markedly less, which may amongst other factors explain the lack of improvement in the single sprint time. The significant improvement seen in HRR within this study was expected, as studies of HR, following endurance training, have shown significantly faster HRR kinetics in both adolescent endurance trained athletes (Ostojic et al., 2010) and young swimmers (Perini et al., 2006). Even though exercise capacity has been linked with an increased HRR (Cole et al., 1999) and exercise training has been shown to improve HRR (Lamberts et al., 2009), correlations between HRR and swim performance are lacking (Costa et al., 2012a; Perini et al., 2006; Atlaoui et al., 2007). Interestingly, within the current study, a significant negative relationship (Fig. 7) was observed between HRR and SP. The explanation for this finding cannot be easily explained and warrants further investigation.

### Implications for swim training programs

High volume aerobic training has been shown to increase muscle oxygen extraction as indicated in this study and in other land based studies (Kime et al., 2010; Neary, Mckenzie & Bhambhani, 2002). Similarly, high volume aerobic training has been shown to improve performance (Costa et al., 2013). Additionally, the strong correlation found between uNIRS measures and performance measures in the current study suggest that high volume aerobic training is the cause of the improved swim performance. A keenly debated area within swim literature is the efficacy of high training volumes, which do not match the energetic demands of swim competition (Libicz, Roels & Millet, 2005; Aspenes & Karlsen, 2012; Maglischo, 2003). It has been shown that exercise intensity (rather than volume) is a key determinant of performance enhancement (Pyne, Lee & Swanwick, 2001; Toubekis et al., 2011). A possible explanation for the lack of further associations between SP and improvements in peripheral and systemic capacity may be related to an improvement in the swim efficiency/movement economy that has previously been shown to differentiate between novice and elite swimmers (Barbosa et al., 2010). An enhanced swim efficiency would be responsible for the improvements in swim velocity, an important determinant of swim performance (Toussaint, 1990; de Mello Vitor & Silveira Böhme, 2010). It may be that the acquisition of this improved skill can only be achieved or “learned” via high volume repetitive swim actions and is therefore a key factor in high volume aerobic swim training (Cunningham & Eynon, 1973). However recent evidence suggests that swimming performance in short distance, high intensity events are metabolic dependent, rather than economy dependent (Ribeiro et al., 2015). However, as changes in swim efficiency and technique were not assessed within the current study an improvement in this capacity could not be determined.

### Limitations

It is important to recognize the inherent limitations within this study and the subsequent effect upon the interpretations of the major findings. No synchronization of movement features/NIRS data took place, as such interpretation and discussion of NIRS swim turn data is based purely on visual analysis. It is possible that given the short duration of the exercise, improvements in other aspects of muscle metabolic control could have occurred. Therefore interpretation of post-training improvements could have been supported by incorporation and analysis of critical velocity and oxygen uptake kinetics, which could provide a valid field of application for uNIRS testing. The decision to choose the *vastus lateralis* muscle only was a practical limitation. This restricted the option to observe the upper body musculature (*deltoid, latissimus dorsi, triceps brachii*) which is considered to be more dominant in swimming (Stirn et al., 2011; Konstantaki, Winter & Swaine, 2008). Examination of these muscle groups may have provided a more appropriate measurement during this type of training. Previous work (Jones & Cooper, 2016) demonstrates that club level swimmers (adults) desaturate the upper (*latissimus dorsi*) and lower (*vastus lateralis*) body to the same degree during a maximal 200 m freestyle swim and the lower limbs have been shown to be recruited to a greater extent during swim sprint distances (50–100 m) (Fulton, Pyne & Burkett, 2009; Maglischo, 2003). Finally, whilst training load, volume and adherence were monitored, no specific focus on training intensity was undertaken. Further research is warranted is in this area.

## Conclusion

The development of the uNIRS technology has enabled for the first time measurement of training induced changes in peripheral muscle hemodynamics and oxygen metabolism within the swim athlete’s primary exercise environment. Historically measurements “in the field” in water-based events have significantly lagged behind land-based activities. We suggest that uNIRS could be used as a complimentary technology in support of sEMG and central measures of oxygen uptake to, in part, redress this imbalance.

## Supplemental Information

10.7717/peerj.4393/supp-1 Supplemental Information 1 Raw data.

## Additional Information and Declarations

### Data Availability

The following information was supplied regarding data availability: All raw (individual) data is provided in the Results section of the manuscript, and is provided as Supplemental Dataset Files.
